# Role of Mechanotransduction and Tension in T Cell Function

**DOI:** 10.3389/fimmu.2018.02638

**Published:** 2018-11-15

**Authors:** Jérémie Rossy, Julia M. Laufer, Daniel F. Legler

**Affiliations:** ^1^Biotechnology Institute Thurgau (BITg) at the University of Konstanz, Kreuzlingen, Switzerland; ^2^Department of Biology, University of Konstanz, Konstanz, Germany

**Keywords:** T cell, mechanotransduction, tension, adhesion, migration, TCR, actin cytoskeleton, signaling

## Abstract

T cell migration from blood to, and within lymphoid organs and tissue, as well as, T cell activation rely on complex biochemical signaling events. But T cell migration and activation also take place in distinct mechanical environments and lead to drastic morphological changes and reorganization of the acto-myosin cytoskeleton. In this review we discuss how adhesion proteins and the T cell receptor act as mechanosensors to translate these mechanical contexts into signaling events. We further discuss how cell tension could bring a significant contribution to the regulation of T cell signaling and function.

## Introduction

To mount a proper adaptive immune response and establish immune memory, T cells carry out many distinct cellular processes. In a simplified view, these processes can be grouped in three categories. (a) The adhesion cascade, during which circulating T cells exit the blood flow to roll, adhere and eventually extravasate through the endothelial cell layer. (b) Migration, on the wall of blood or lymph vessels, within lymph nodes and inflamed or cancerous tissues. And (c), activation, which primes naïve T cells and triggers cytotoxicity and cytokine secretion from effector cells. The molecular interactions and signaling pathways associated with T cell activation (1), migration through venular walls (2) and T cell migration in general (3) have been extensively characterized and are comprehensively described in these recent reviews. But the emergence of novel biophysical approaches has allowed to shine light on a previously neglected aspect of these processes: they all generate mechanical stimuli.

During the adhesion cascade, the blood flow applies an external shear stress on T cells binding and migrating on and through endothelial cells (2). T cell migration in tissues is driven by morphological changes, constantly fluctuating actin polymerization and molecular motors-driven contractions, which all generate internal mechanical tension (4). It goes the same with T cell activation, which involves a tight contact between T cells and antigen-presenting cells or target cells, acto-myosin contractions and a sustained actin retrograde flow (5). Adding to the multiplicity of these mechanical contexts, T cells interact with substrates displaying various and changing stiffness (6) and with adhesion molecules that are either diffusive or firmly anchored to cortical actin (7). Hence, the idea that force plays an essential role in the T cell-mediated immune response has matured from an exciting hypothesis to a well-established field of T cell biology (8–11).

In this review we first focus on demonstrated mechanotransduction events in T cells. We discuss how adhesion proteins—selectins and integrins—and the T cell receptor (TCR) act as mechanosensors during the adhesion cascade and during T cell activation, respectively. In the second part of the review, we get inspiration from other cell types and systems to picture how cell tension might contribute to the cellular signaling that regulates T cell migration and activation.

## Shear force: a key player during T cell rolling and arrest on the endothelium

**Figure 1:**
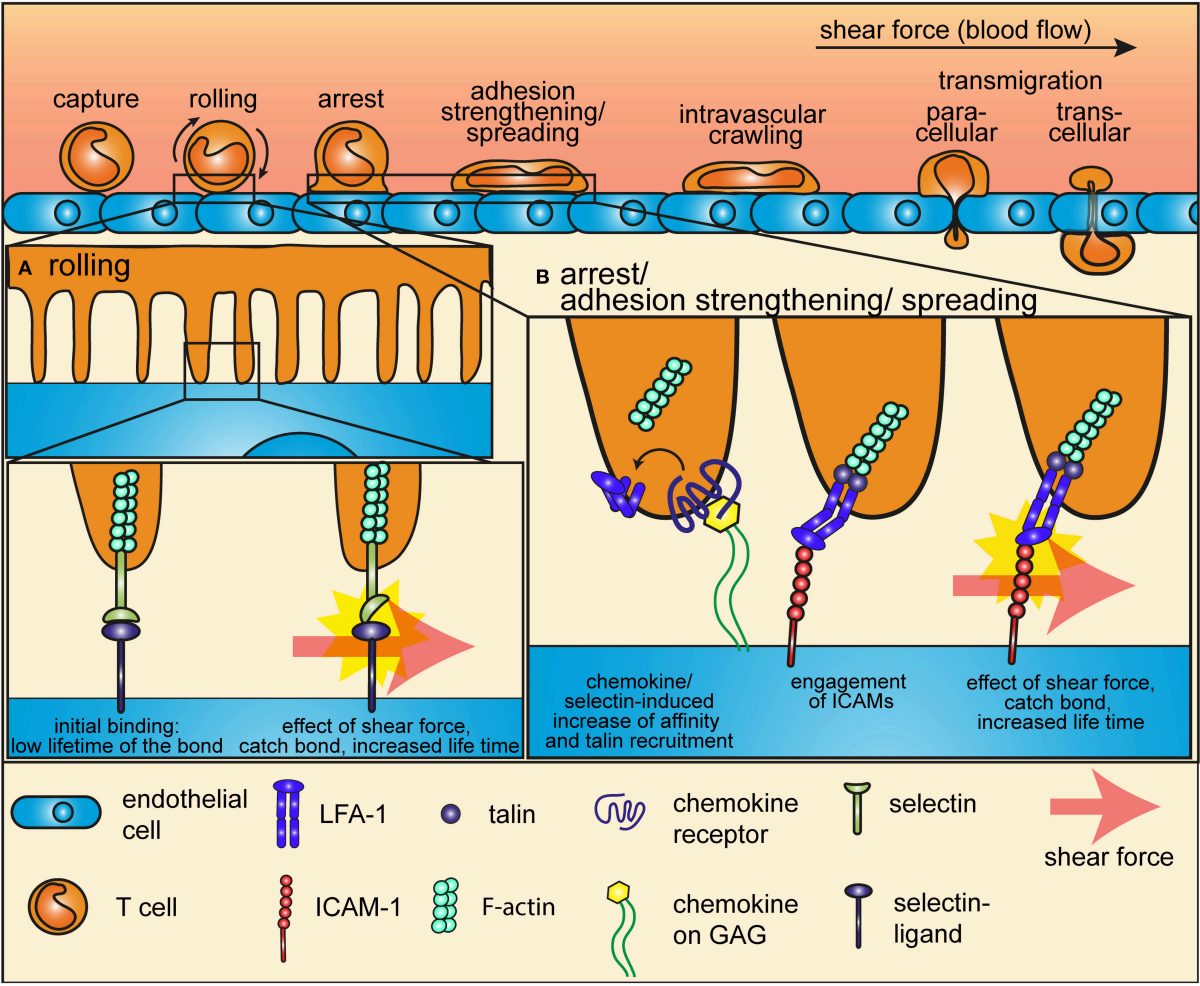
Mechanotransduction during the adhesion cascade. The proteins that mediate rolling and arrest of T cells on endothelial cells during the adhesion cascade are mechanosensors, which are sensitive to and relies on the force of the shear flow. **(A)** During the early steps of the adhesion cascade, selectins at the tip of microvilli of T cells interact with their ligands at the surface of endothelial cells to mediate tethering and rolling. Shear force impose a tension on this bond and thereby induces a conformational change in the selectin headpiece, which gives to the selectin-ligand bond a catch-bond characteristic. **(B)** Integrins mediate arrest after rolling and firm adhesion to the endothelium. Shear force also plays an essential role in this multistep process. Inside-out signaling from selectins of from chemokine receptors induces a first conformational change that increases the affinity of integrins for ICAMs and anchors them to the cytoskeleton through the recruitment of talin. Shear force pulls ligand-bond integrins into a high affinity, open conformation and increases the life-time of the bond through a catch-bond process.

In search for their cognate antigen, T cells circulate between peripheral tissues and secondary lymphoid tissues, thereby exploiting a network of blood and lymphatic vessels (12). T cells circulating in the blood enter lymph nodes through high endothelial venules (HEVs). Before they can extravasate trough HEVs, T cells first need to roll, arrest and finally adhere to the vessel walls (2, 13). Forces derived from the blood flow play a decisive role in this adhesion cascade, contributing both to the initial capture by selectins and to the firm integrin-mediated arrest preceding extravasation (Figure 1).

Rolling on HEVs is mediated by fast on and off rates interactions between selectins on T cells and their ligands displayed by the endothelium. Pioneering work using atomic force microscopy (AFM) in combination with flow-chambers revealed that selectin-ligand interactions form catch bonds—molecular interactions whose dissociation rate decreases with force, see Glossary at the end of the article—when subjected to low shear force generated by the blood flow (11, 14, 15). Thus, a mechanotransduction process, driven by a conformational change in the selectin headpiece, prolongs the life time of the bond between selectins and their ligand and thereby gives rise to enhanced cell adhesion under flow conditions (Figure 1A).

T cell tethering and rolling eventually leads to arrest and firm adhesion on endothelial cells, which is driven by heterodimeric integrins and their ligands and which also requires low force from the blood flow (2, 13, 16). Remarkably, integrin adhesiveness is increased very shortly after T cells make contact with endothelial cells, through a multistep process during which force plays an essential role (17). The first step in integrin-mediated adhesion is activation by signals coming from selectins and chemokine receptors. In a certain way, this first step prepares integrin to bear tensile forces, as it (a) increases integrin affinity for immobilized ligands on the extracellular side and (b) strengthened integrin-actin cytoskeleton connection on the intracellular side through the recruitment of talin and kindlin to the intracellular integrin tail (17, 18). Indeed, integrin activation by chemokines alone is not sufficient to trigger adhesiveness, which is achieved only by the effect of shear force from the blood flow (19). Integrins bound to immobilized ligand on one side and firmly anchored to the actin cytoskeleton on the other side are pulled into a high affinity, open conformation by the low force of the shear flow (Figure 1B). This force-mediated reorganization of integrin conformation eventually allows stable bonds with ligands at the surface of endothelial cells to support T cell immobilization.

## T cell migration: steering toward stiffness

After adhesion and extravasation through endothelial cells, T cells adopt a motile behavior to reach antigen-presenting cells in lymph nodes or inflamed tissues. As described in an excellent recent review, the link between the actin cytoskeleton, adhesion modules and the extracellular matrix is highly dynamic and allows cells to convert the mechanical properties of their environment into signaling (20). In the context of migration, this can result in durotaxis—the ability of cells to migrate toward stiffer substrates. Durotaxis is another way how mechanotransduction could potentially contribute to T cell functions. Typical targets of T cells, such as (a) cancer cells that can be softer than normal cells (21); (b) tumors, that are stiffer than normal tissue because of high collagen density and crosslinking (22, 23), or (c) antigen-presenting cells (6) have specific stiffness properties. Changes in extracellular matrix stiffness of specific tissues are generally associated with disease progression (24). Neutrophils, whose amoeboid type of migration is similar to that of T cells, spread more and migrate slower but more persistently and exert stronger traction forces on stiffer substrates (25, 26). Like neutrophils, T cell migration on ICAM-1 coated surfaces is also influenced by substrate rigidity. Indeed, it has been recently shown that T cells migrate faster on stiffer substrates (27).

## T cell activation needs force

**Figure 2:**
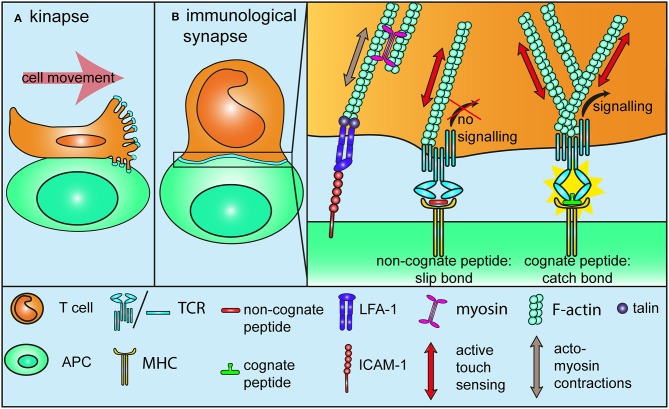
Mechanotransduction during T cell activation. **(A)** TCRs at the tip of microvilli are subjected to specific forces while T cells migrate on or form a kinapse with antigen-presenting cells. It is not yet determined if tensions are “absorbed” due to the elastic nature of microvilli, or if on the contrary, microvilli push against the antigen-presenting cells, thereby increasing the tension on TCR **(B)** During and after the formation of an immunological synapse with a cell presenting a cognate antigen, migration-related, and acto-myosin-mediated tensions drive integrins into a full affinity state, similarly to what happens during the adhesion cascade (Figure 1B). These forces also lead to passive mechanosensing by TCR. Additionally, TCR itself further engages in active mechanosensing, by pulling and pushing on pMHC molecules. Non-stimulatory ligands form slip-bonds under tension and fail to trigger TCR signaling. By contrast, stimulatory ligands engage in a catch-bond with TCR, which leads to a conformational change and in turn promotes TCR signaling. Binding to a stimulatory ligand also increase the density of F-actin around the TCR to further anchor it to the underlying cytoskeleton. All in all, tensions through the TCR-pMHC bond contribute to TCR triggering and antigen discrimination.

Contact of a migrating T cell with a target cell or an antigen-presenting cell displaying a cognate antigen result in activation and arrest and in the formation of an immunological synapse (1, 28). In this paragraph, we will discuss in detail how mechanotransduction plays an essential role in this process. By demonstrating that T cell activation with antigen-coated beads requires the beads to be larger than 4 μm, Mescher provided the first hint that the generation of tension over a significant scale is indispensable for T cell activation (29). The first mechanosensor model for TCR was published quite some time later, in a study demonstrating that the binding of an immobilized agonist antibody to CD3ε induces a torque in the structure of the TCR-CD3 complex. Non-activating antibodies however, need to be conjugated to a bead and pulled tangentially to the receptor using optical tweezers to induce a similar activating response (30, 31). By suggesting that the migration-related movement of T cells engaging a cognate peptide at the surface of antigen-presenting cells induces tangential forces on TCR, this study is also an important reminder that T cells are actually migrating and under tension when they find their cognate antigen. Mechanosensing cells or proteins can sense and react to externally applied mechanical stimuli, without actively contributing to the force that is at the source of the stimulus. For instance, in the case of a cell submitted to shear stress. This can be termed passive mechanosensing (32), in contrast to active touch sensing (mentioned further in this review), where the mechanosensor is actively involved in the mechanical stimulus it is sensitive to, a bit like poking a mango to determine if it is ripe or not. Cell motility generates cell tension and thereby might lead to passive mechanosensing as migration-related forces are transferred onto the TCR-CD3 complex (Figure 2A). Similarly, formation of the immunological synapse leads to activation of the integrin LFA-1 and to tight adhesion to immobilized ICAM-1 on antigen-presenting cells (33), as well as, acto-myosin contractions (34) and cytoskeletal tensions (35, Figure 2B). Hence, transition from migration to activation upon engagement of a cognate peptide represents a mechanical signal that is very likely to results in passive mechanosensing by TCR. Interestingly, TCR engagement promotes local actin polymerization around the receptor itself (35), in a way that reminds of the signal-dependent and talin-mediated anchorage of integrins to the actin cytoskeleton during the adhesion cascade. This means that TCR is further anchored to the underlying cortical actin cytoskeleton upon activation, which could very well make it more susceptible to respond to mechanical stimuli. Along this line, it is now well-established that T cells, like many other cells, engage in the “active touch sensing” described by Kobayashi and Sokabe (32) by actively pushing and pulling on the substrate they adhere to in order to interrogate its stiffness (Figure 2B). Within the first tens of seconds of TCR triggering on a biomembrane force probe setup, T cells engage in a sequence of pushing and pulling forces even in the absence of LFA-1 engagement (36). Traction force microscopy (TFM) on polyacrylamide gels further confirmed that antibody activation of CD3 leads to acto-myosin-mediated pulling forces, which originate at the cell edge and are directed toward the cell center (37). Another TFM study on micropillars determined that these centripetal forces are generated through the binding of TCR to activating ligands, further suggesting that integrins are not the mechanosensor at play during T cell activation (38). These forces are in the range of 100 pN, which is lower than the nanonewton forces observed during epithelial cells migration (39). Of note, phosphorylation of the early TCR signaling kinase Lck takes place on the side of the pillars facing the cell edge, suggesting that TCR signaling is triggered where the tension is highest and strengthening the idea that TCR works better when it is under tension (38). The surface of T cells is covered with microvilli, whose tips are enriched with TCR [(40, 41), Figure 2A)]. These microvilli extend and retract while T cells scan antigen-presenting cells and it is likely that the first step of antigen recognition on antigen-presenting cells is mediated by TCR located on stretched microvilli. This raises the possibility that active touch sensing might already be involved in the very early stages of T cell activation, as TCR at the tip of microvilli is subjected to specific forces resulting from the scanning of antigen-presenting cells. But forces applied on TCR at the tip of microvilli are also likely to be reduced by the elastic nature of these projections, which can act as shock absorbers, for instance in the context of the adhesion cascade (42). Further investigations are required to determine if TCR at the tip of microvilli is put under tension due to the exploratory character of these projections, or if on the contrary, the force on TCR is dissipated through a shock absorber effect. Finally, forces imposed on TCR located on collapsed microvilli will be very different once the immunological synapse is fully established.

A direct consequence of the active touch sensing through TCR is that T cell activation is influenced by substrate stiffness. As a matter of fact, T cells pull more on stiffer substrates than on softer ones (37). CD4 T cells produce also more IL-2 on harder substrates up to 100 kPa (27, 43), but the stiffness contribution to T cell activation is somehow lost beyond 100 kPa (43, 44). More generally, every aspect of T cell activation is potentiated by stiffer surfaces up to 100 kPa (27). The effect of substrate stiffness on T cell activation could even be larger than reported in these studies, which all used functional antibodies against CD3 to activate T cells. It is indeed likely that differences in the rigidity of substrates might have a more pronounced effect on the binding of TCR to its natural ligand, a cognate peptide presented by major histocompatibility complex (MHC), than to an activating antibody.

The mechanism behind stiffness sensing in T cells is not identified yet, but talin might be involved. As part of the complex protein assembly between integrins and the actin cytoskeleton (45), talin is an essential element of the substrate stiffness sensing machinery and preventing talin to mechanically engage with integrin disrupts extracellular rigidity sensing (46). Interestingly, T cells lacking talin fail to stop migrating in response to TCR triggering (47). As mentioned above, talin is essential to integrin-mediated adhesion (17) and in particular to LFA-1 adhesiveness for ICAM-1 following TCR triggering (48). It is likely that the affinity of LFA-1 for ICAM-1 is increased during T cell arrest upon TCR activation through a similar mechanism than described above during the arrest on endothelial cells in the blood flow. One can indeed consider that during activation, the LFA-1—ICAM-1 bond is put under tension by acto-myosin contractions and actin retrograde flow in a similar fashion that it is stretched by extracellular forces resulting from shear flow during the adhesion cascade (Figure 2B). As a matter of fact it has been shown that ICAM-1 is immobilized at the surface of antigen-presenting cells in order to promote T cell-antigen presenting cells conjugation and T cell activation (33). Hence talin mechanosensing properties could contribute to the stop signal that precedes the establishment of the immunological synapse and eventually to full T cell activation. However, a recent study somehow challenges the idea that the talin-LFA-1 axis supports the stop signal. Feigelson et al. reported that the integrin ligands on antigen-presenting cells, ICAM-1 and -2, are dispensable for these cells trigger arrest activation of T cells (49). Finally, intravital microscopy studies have shown that T cells do not necessarily stop when encountering a stimulatory antigen-presenting cells. Antigen recognition can happen during long-lasting contact, the immunological synapse, but also during shorter and more dynamic interactions, termed *kinapse* (28, 50, Figure 2A). While the functional difference between synapse and kinapse has not been fully established, the duration and nature of the antigen-presenting cell-T cell interaction contribute to shape the outcome of T cell activation (51). Therefore, it is likely that the mechanosensitive properties of integrin and TCR contribute to this process by leading to distinct signaling in the context of a synapse or of a kinapse.

Thus, T cells pull on activating substrates and they are more susceptible to be activated by stiffer substrates. Having this in mind, it does not take a bit leap to imagine that the active touch used by T cells is not only a mechanism to interrogate substrate stiffness. Indeed, a few recent studies indicate that putting TCR under tension is in fact an integral part of the activation process (Figure 2B). Presenting T cells with activating peptide-MHC complex (pMHC) on an AFM microscope showed that T cell activation requires both the binding of a cognate antigen and forces through TCR (52). An in depth analysis of the kinetics of TCR-pMHC interactions using a biomembrane force probe showed that TCR establishes catch bonds with cognate pMHC and slip bonds—molecular interactions whose dissociation rate increases with force—with non-agonistic pMHC, thereby making force applied through TCR a component of the antigen discrimination process (53). The formation of catch bond is even what distinguishes stimulatory from non-stimulatory ligands between peptides that bind TCR with similar affinity (54). These results are further confirmed by two studies from Lang and colleagues using optical tweezers and DNA tethers. They first identified an elongated structural element of the TCRβ constant chain, the FG loop (55), as a key factor for the contribution of the force in antigen discrimination (56). More recently, they demonstrated that TCR needs non-physiological levels of pMHC molecules to be triggered in the absence of forces (57). Using DNA-based nanoparticle tension sensors Liu et al. further demonstrated that piconewton forces are transmitted through TCR-CD3 complexes a few seconds after activation and that these forces are required for antigen discrimination (58).

In summary, passive mechanosensing of the forces resulting from migration and activation, and active touch sensing through the TCR-CD3 complex probably act together to connect TCR triggering at the same time to the physical environment (speed of migration, stiffness of the presenting cells) the T cell evolves in and to ligand selectivity (8). This maybe brings us back to a model described just 10 years ago, which proposed that the TCR-CD3 complex requires to be stretched in order to be activated (59). A postulate that is strengthened by the fact that TCR triggering involves a mechanical switch of its structure (60).

Forces that T cells generate upon activation do not relate only to signal intensity and specificity, but also contribute to the T cell response, notably in the context of killing. Cancer target cells that express a higher number of adhesion molecules facilitate the release of lytic granules by cytotoxic T lymphocytes (61). More strikingly, tension induced on target cells by cytotoxic T lymphocyte facilitates perforin pore formation in target cells and thereby increases the transfer of granzyme proteases and cytotoxicity (62).

## Tension in T cells: further facts and perspectives

Cell tension is the result of a complex interplay between tension mediated through the cytoskeleton and membrane tension. The cortical actin—plasma membrane relationship plays a central role in mechanobiology and is very well described in recent reviews (63, 64). In this regard, proteins that link the plasma membrane to the underlying cortical actin such as Ezrin/Radixin/Moesin (65) are likely to play a determining role in T cell mechanical properties and mechanotransduction. Ezrin, which directly regulates membrane tension (66) is deactivated upon T cell activation to promote cell relaxation and *in fine* conjugation to antigen-presenting cells (67). Similarly, constitutively active Ezrin increases membrane tension and impairs T cell migration *in vivo* (68). Hence, it appears that the ability of T cells to relax and deform their membrane is directly related to their ability to migrate and be activated. This is confirmed by the fact that naïve T cells are less deformable than T lymphoblasts, as assessed by a micropipette aspiration assay. The same study showed that depolymerization of the actin cytoskeleton makes naïve T cells and T lymphoblasts more deformable altogether (69).

Variations in membrane tension can influence T cell signaling in various ways. Mechanosensitive (MS) channels open up to mediate ion flux in response to membrane stretch (32, 70). First discovered in bacteria where they compensate for sudden changes in environmental osmolality, MS channels have been shown to mediate intracellular Ca^2+^ rise in response to tension applied to focal adhesion or along actin fibers (71). T cells express a large variety of potential MS channels (72) and an electrophysiological study showed that one of them, TRPV2, opens and mediates Ca^2+^ entry in T cells subjected to mechanical stress (73). It has recently been shown that the most potent mechanosensitive ion channel identified to date, Piezo 1, is expressed in T cells, where it contributes to T cell activation through Ca^2+^-influx, albeit the study did not actually investigate if this is through mechanical stress (74). In this regard, a study using AFM in synchronization with fluorescence imaging reported that mechanical stimulation alone, without TCR stimulation, is sufficient to elicit an increase in intracellular Ca^2+^ (75). This is in agreement with the expression of Piezo 1 in T cells, but somehow in contradiction with Hu and Butte, who reported that mechanical stimulation triggers Ca^2+^ flux only when coupled with TCR triggering (52). Further studies are still required to determine whether or not mechanical stimuli alone are sufficient to trigger Ca^2+^ flux through Piezo 1 in T cells.

Whether or not MS channels play a role in T cell migration also remains to be determined. It is however likely that membrane tension contributes to organize polarity during T cell migration, in light of what has been observed in neutrophils. Ten years after the inhibitory effect of cell tension on the small GTPase Rac had been shown (76), Houk et al used micropipette aspiration to show that cell tension acts as a long-range inhibitor to prevent Rac-mediated actin protrusions elsewhere than at the leading edge of motile neutrophils (77). These results were extended to further demonstrate that cell tension limits actin assembly through a negative feedback pathway involving phospholipase D2 and the mammalian target of rapamycin complex 2 (mTORC2) (78). Membrane tension also impact on the distribution and dynamics of membrane-bending proteins, such as BAR domain proteins (79), and reciprocally (80). In this context, it is interesting to note that tension promotes membrane tensformation of the leading edge of COS-1 cells, through the recruitment of FBP17, a membrane-bending and curvature-sensing activator of WASP-dependent actin polymerization (81). Even though T cells and COS-1 cells have noticeably different mechanisms of migration, it seems likely that tension and actin polymerization could act in concert to install polarity in migrating and in activated T cells *via* similar mechanisms.

Carrying the speculation further, we could even imagine that the contribution of membrane tension to T cell activation or migration extends to the regulation of intracellular trafficking. As discussed in comprehensive reviews, the plasma membrane is largely inelastic and can increase in area only 2–3% before rupture occurs (63, 82, 83). Consequently, cells actively respond to membrane tension through regulation of intracellular trafficking, increased membrane tension favoring exocytosis (84–86) and reduced membrane tension leading to endocytosis (87). This means that cell tension could act as a mechanical long-range messenger to directly influence and coordinate endocytic and exocytic events (82, 83, 88) taking place during T cell migration and activation. In fact, intracellular trafficking is a key factor in establishing functional polarity by spatially restricting membrane proteins at a specific localization in the cell, thereby confining signaling and interactions with other cells or with the extracellular matrix. Selective endocytosis of a given receptor can locally reduce its surface expression. Similarly, targeted recycling can increase the local concentration of a protein within the plasma membrane. Incidentally, T cells are highly polarized, both during migration (uropod vs. leading edge) and during activation (immunological synapse). It is thus possible that membrane tension contributes to the regulation of these processes through the organization of specific endocytic and exocytic events. For instance, endocytosis and recycling are essential to integrin polarization and activity in motile cells in general (89, 90) and in T cells in particular (91, 92). Similarly, targeted delivery of vesicles to the immunological synapse is required for full T cell activation (93, 94) and secretion of cytotoxic granules (95, 96). A good illustration of how this could happen can be found during phagocytosis by macrophages, a process that is in many ways similar to the formation of the immunological synapse and during which membrane tension coordinates the actin-driven formation of the phagocytic cup and exocytosis-fusion of vesicles (97).

Finally, cell tension does not stop at the plasma membrane or the cortical cytoskeleton. As well described in a recent review, forces are transferred from the cell surface to the nuclear envelope through the intermediate of the cytoskeleton or directly from the external environment (98). The structure and function of the nucleus are affected by these tensions, which allows it to function as a mechanosensor (99, 100). Accordingly, tensions can regulate gene expression by modifying the connection of heterochromatin to the nuclear lamina (101). Forces transferred to the nuclear envelope have also been reported to favor cell proliferation (98). Nuclear deformation has further been shown to directly lead to the import of specific transcription factors through the opening of nuclear pore complexes (102, 103). Because of its size and rigidity, the nucleus is the limiting factor during cell migration in a dense meshwork (104). Typically, dendritic cells use myosin II-driven contractions (105) and produce a dense actin network around the nucleus (106) to promote nucleus deformation and in turn facilitate squeezing through constrictions. 3D migration of T cells in confined environments is thus very likely to lead to compression of the nucleus. Similarly, the pulling exerted by T cells on antigen-presenting cells is susceptible to lead to compression or even flattening of the nuclear envelope. Hence it is conceivable that tension resulting from prolonged migration in confined environment or from T cell binding to an antigen-presenting cell can lead to rearrangement of the chromatin structure or to the opening of nuclear pores and thereby influence the regulation of gene expression leading to T cell differentiation or proliferation.

## Conclusion

**Figure 3:**
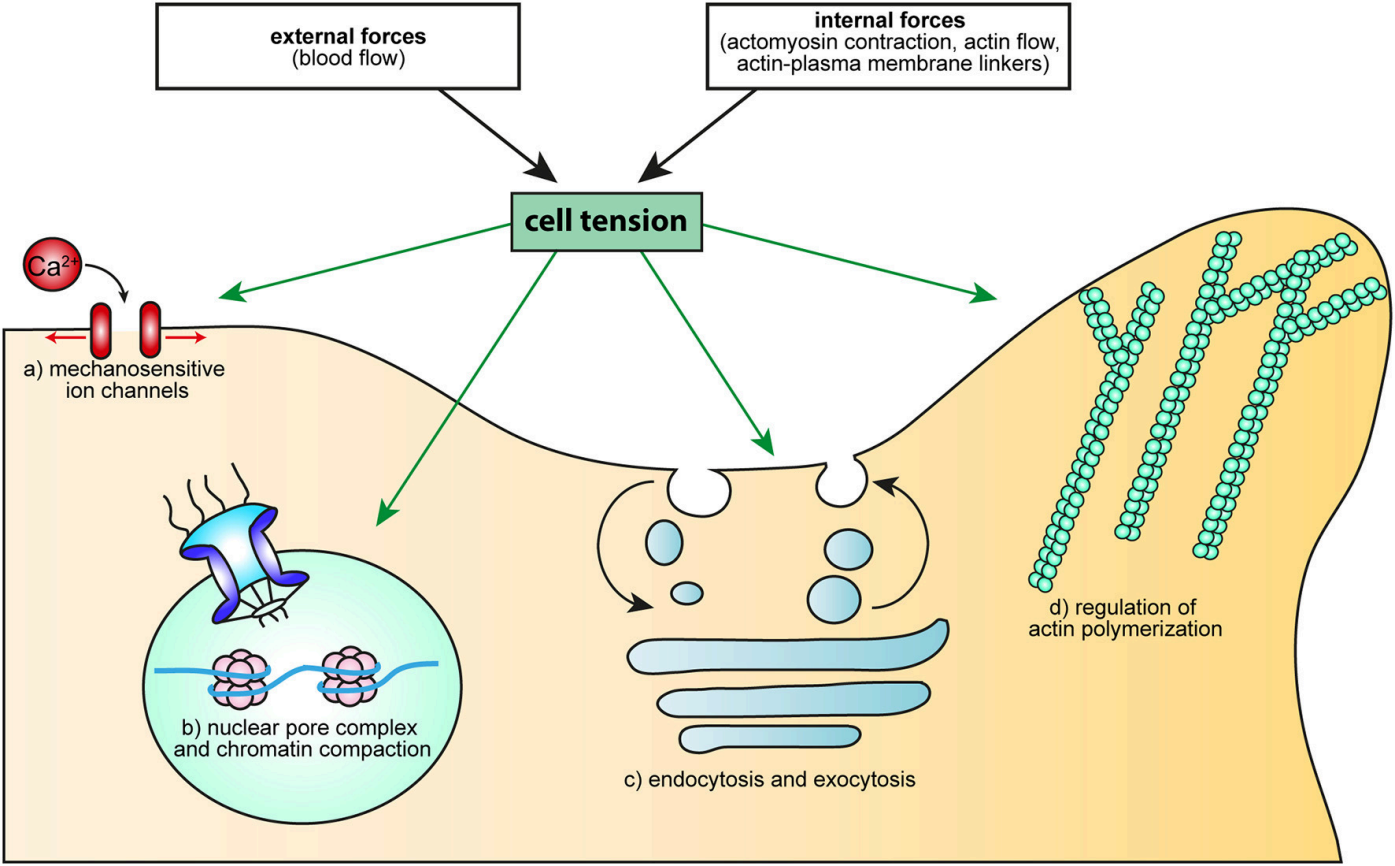
How cell tension could regulate cellular processes essential to T cell activation. Cell tension, either generated by external forces (such as the blood flow), or resulting from intracellular mechanisms (molecular motors, actin flow, modification of the linkage between membrane and the cytoskeleton) can regulate: (a) Ca^2+^ flux, through MS ion channels, (b) cell proliferation and differentiation, through the opening of the nuclear pore complex or modification of chromatin compaction, (c) endo- and exocytosis, *via* membrane tension and d) actin polymerization, through the activity of the small GTPase Rac or the binding and unbinding of BAR domain proteins.

T cells are subjected to ever-changing forces, either generated intracellularly or from their environment. They further interact tightly with cells displaying various levels of stiffness and with molecules whose anchorage to the underlying actin cytoskeleton varies. But more important than the multiplicity of these mechanical contexts, is the fact that they very often are associated with specific processes participating to T cell function. It is therefore very likely that distinct mechanical signals team up with biochemical signals to ensure that T cells do the right thing at the right place and time. The role of mechanotransduction in the adhesion cascade preceding extravasation and in T cell activation is now well-established, although there is still room to refine the model describing it. Now is maybe the time to investigate the importance of cell tension for T cells (Figure 3), using what we have learned from other cell types and taking advantage of ever-improving biophysical approaches.

## Author contributions

JR and DL conceived and wrote the manuscript, JL draw the illustrations.

### Conflict of interest statement

The authors declare that the research was conducted in the absence of any commercial or financial relationships that could be construed as a potential conflict of interest. The handling editor declared a past co-authorship with the authors DL and JL.

## Glossary

**Table:**

Shear stress (or shear force)	Shear stress is the tangential force applied by a flowing fluid on the surface of an object
Catch bond/Slip bond	A catch bond is a bond that becomes stronger (increased lifetime) when a pulling force is applied to it. By contrast, a slip bond becomes weaker (decreased lifetime) with applied force
Mechanosensing	Mechanosensing is the process through which cells or proteins detect and respond to variations of forces and mechanical properties of their environment
Mechanosensor	A mechanosensor is a molecule/protein that mediates mechanosensing
Passive mechanosensing	During passive mechanosensing, a mechanosensor detects applied mechanical stimuli (without applying force or tension itself)
Active touch sensing	Active touch sensing is the process through which cells actively probe the mechanical properties of their environment (for instance substrate stiffness)
Mechanotransduction	Mechanotransduction is the process during which mechanosensors translate mechanical inputs into intracellular signaling events
Stiffness (or elastic modulus)	Stiffness is a measure of the ability of an object or a substance to resist deformation upon an applied force
Durotaxis	Migrating cells can sense stiffness of the substrate they migrate in or on, typically *via* active touch sensing. Durotaxis is the ability of cells to move up rigidity gradients.
